# Supplementary material for: Inner Surface Hydrophilic Modification of PVDF Membrane with Tea Polyphenols/Silica Composite Coating
Source: Polymers (Basel). 2021 Nov 30;13(23):4186. doi: 10.3390/polym13234186 (PMC8659430; doi:10.3390/polym13234186)
Supplement: Supplementary file 1 [file polymers-13-04186-s001.zip › polymers-1472523-supplementary.pdf]

Support information

Inner surface hydrophilic modification of PVDF membrane with tea polyphenols/silica composite coating

Qiang Xu [a], Xiaoli Ji [a], Jiaying Tian [a], Xiaogang Jin [a,*], Lili Wu [a,b,*]

aSchool of Materials Science and Engineering, Wuhan University of Technology, Wuhan 430070
bWuhan University of Technology, Advanced Engineering Technology Research Institute of Zhongshan City, Xiangxing Road 6, Zhongshan, Guangdong, China, 528400
qiangxu@whut.edu.cn(Q.X.); jxl@whut.edu.cn(X.J.); lovertjy@163.com(J.T.)
*Correspondence: polym_jx@whut.edu.cn(X.J.), polym_wl@whut.edu.cn (L.W.)

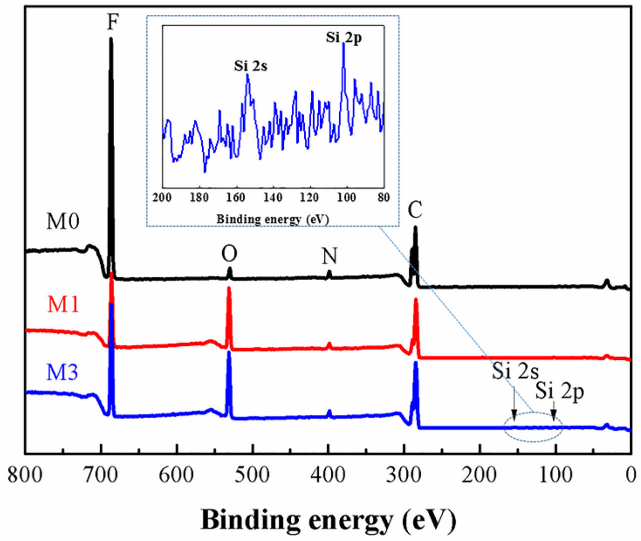

Figure. S1. XPS spectra of modified membrane

Table S1. Elemental compositions of different membranes

Membrane	Composition (at%)				
	C	N	O	F	Si
M0	52.11	2.52	2.67	42.70	/
M1	61.28	3.46	19.36	15.90	/
M3	60.26	3.53	23.27	12.17	0.77

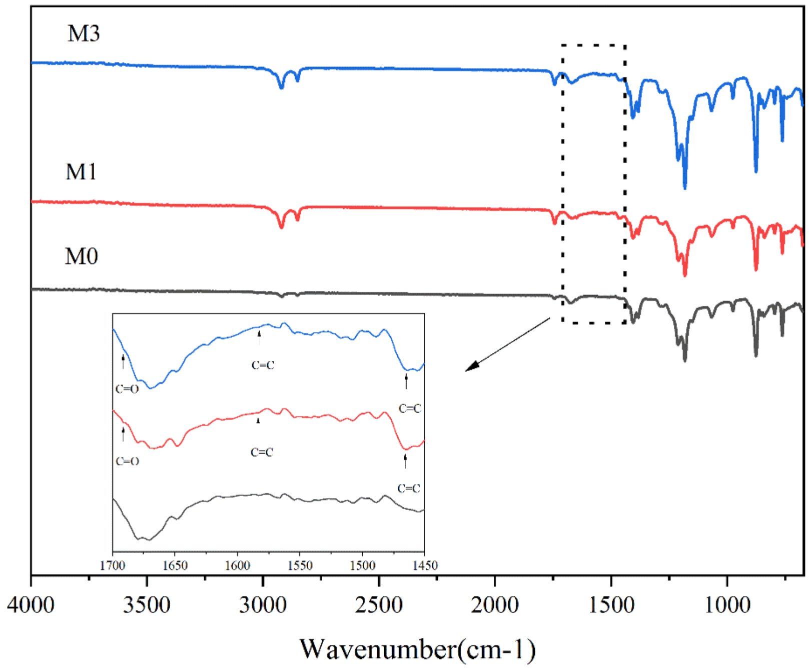

Figure S2. ATR-FTIR spectra of M0, M1 and M3.

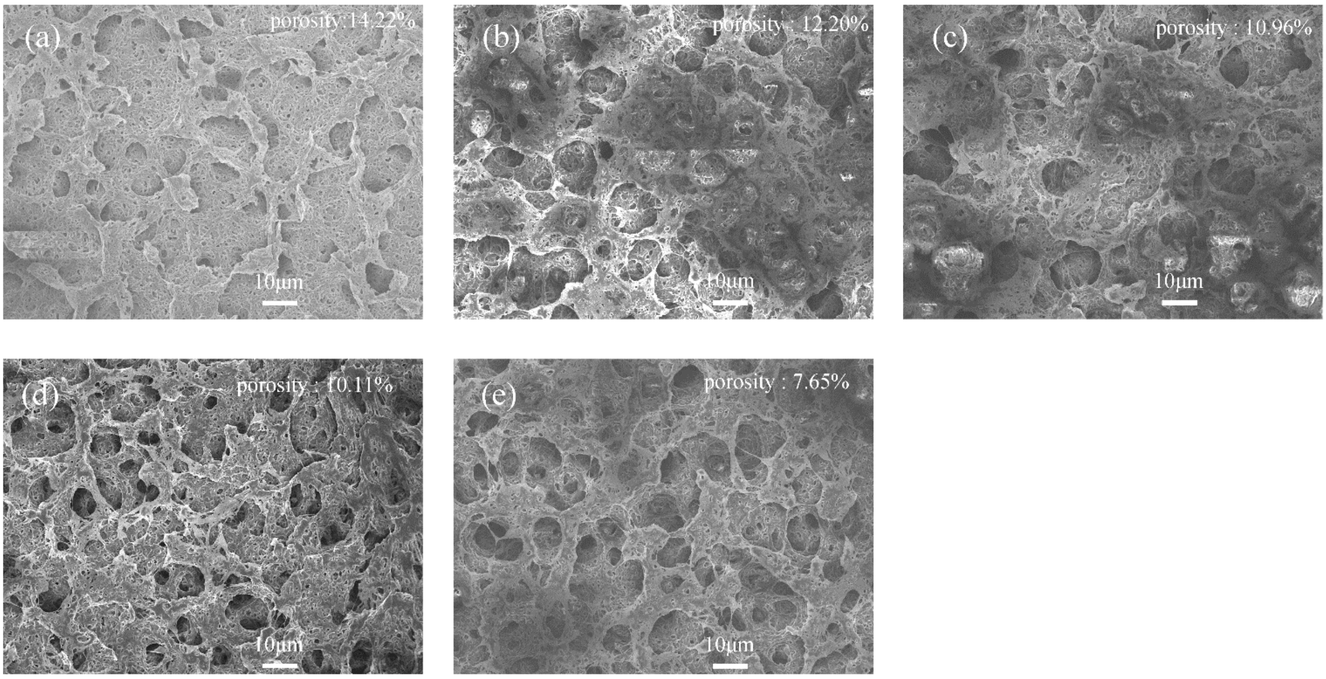

Figure S3. SEM images of the modified membrane surface: (a) M0, (b) M1, (c) M2, (d) M3, (e) M4.

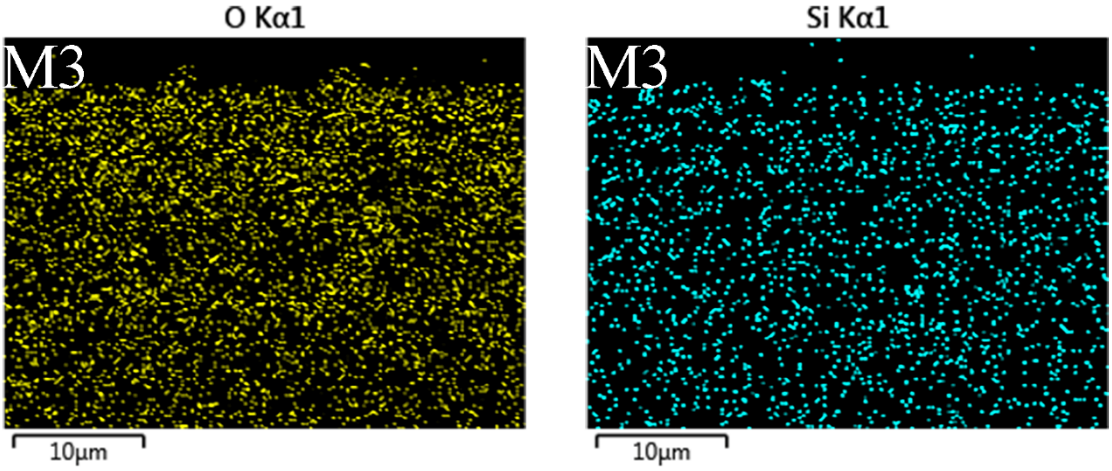

Figure S4. EDX images of the cross-section of modified membrane

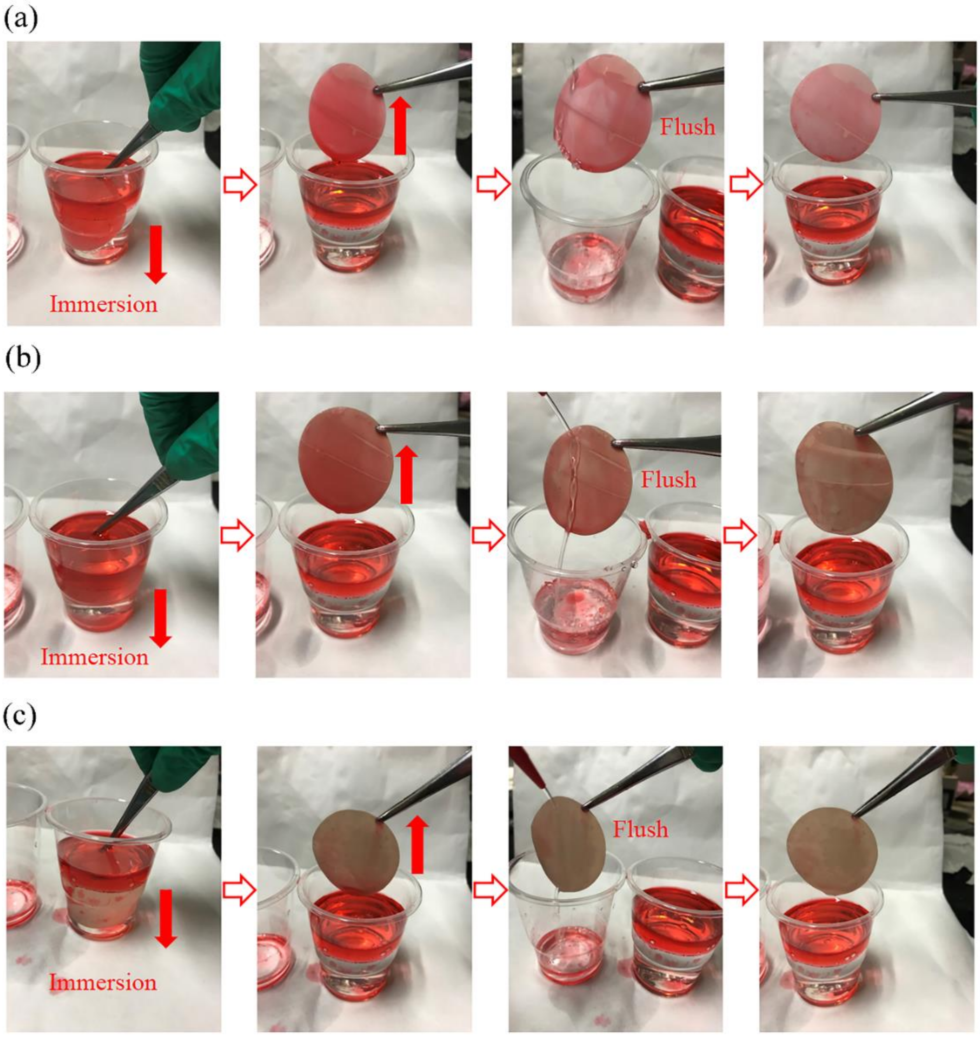

Figure S5. The anti-oil-adhesion property of modified membrane: (a) M0, (b) M1, (c) M3.

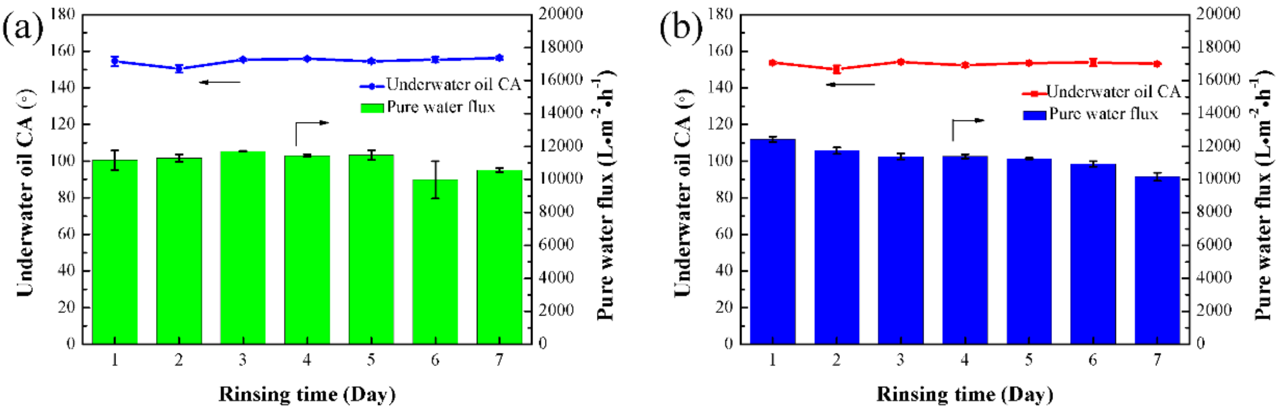

Figure S6. Stability of PVDF-TP film：(a) pH=3，（b）pH=7

It can be seen from Figure S6 that in an acidic environment, the water flux and underwater oil contact angle of the film did not change significantly within 7 days, indicating that the coating is very stable under acidic conditions without additional chemical reaction occurs, and the coating will not easily fall off the surface of the film.

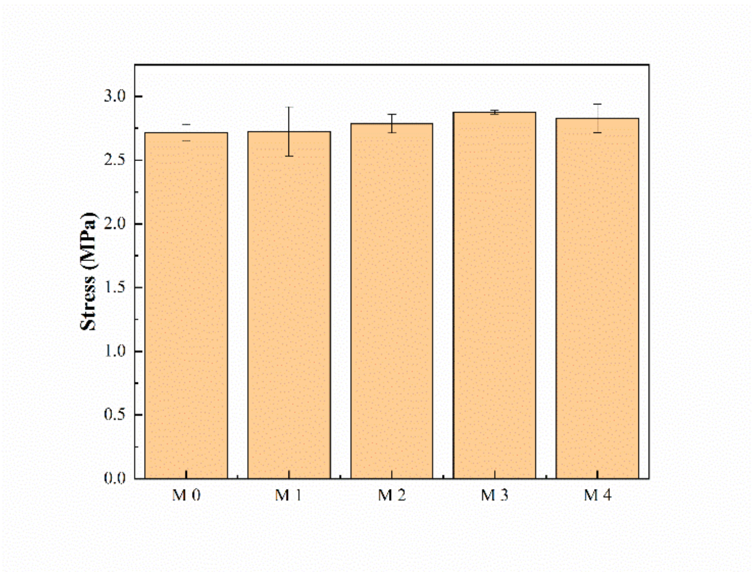

Figure S7. Tensile strength of M0, M1, M2, M3 and M4

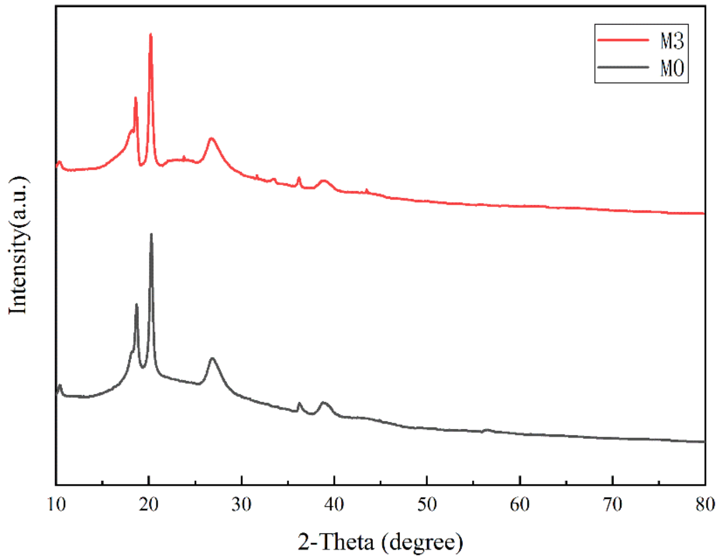

Figure S8. XRD patterns of M0 and M3

The results of Figure S7 show that the tensile strength of the filter membrane hardly changes before and after modification. At the same time, it can be seen from the XRD pattern of Figure S8 that the diffraction peak intensities of M0 and M3 are almost the same. The relative crystallinity of both of them is about 20% by jade software simulation. It can be seen that the mechanical properties of the filter membrane have not changed significantly after being modified.
